# Supplementary material for: Combined crystallographic fragment screening and deep mutational scanning enable discovery of Zika virus NS2B-NS3 protease inhibitors
Source: Nat Commun. 2025 Oct 8;16:8930. doi: 10.1038/s41467-025-63602-z (PMC12508225; doi:10.1038/s41467-025-63602-z)
Supplement: Supplementary file 1 — Supplementary Information [file 41467_2025_63602_MOESM1_ESM.pdf]

Supplementary Information

Combined crystallographic fragment screening and deep mutational scanning enable discovery of Zika virus NS2B-NS3 protease inhibitors

Xiaomin Ni[1†]*, R. Blake Richardson[2†], Andre Schutzer Godoy[3], Matteo P. Ferla[1], Caroline Kikawa[4,5,6], Jenke Scheen[7], William W Hannon[8,9], Eda Capkin[10,11], Noa Lahav[12], Blake H Balcomb[10,11], Peter G Marples[10,11], Michael Fairhead[1], SiYi Wang[1], Eleanor P Williams[1], Charles W.E. Tomlinson[10,11], Jasmin C. Aschenbrenner[10,11], Ryan M Lithgo[10,11], Max Winokan[10,11], Charline Giroud[1], Isabela Dolci[3], Rafaela Sachetto Fernandes[3], Glaucius Oliva[3], Anu V Chandran[10,11], Mary-Ann Xavier[10,11], Martin A Walsh[10,11], Warren Thompson[10,11], Jesse D Bloom[6,13],Nathaniel T. Kenton[14], Alpha A. Lee[14], Annette von Delft[1], Haim Barr[12], Karla Kirkegaard[15,16], Lizbé Koekemoer[1], Daren Fearon[10,11], Matthew J. Evans[2]*, Frank von Delft[1,10,11,17]*

Supplementary Table 1. Data collection and refinement statistics

Protein	ZIKV NS2B-NS3	ZIKV NS2B-NS3 ASAP-0022538
PDB accession code	8PN6	7I9O
Data Collection		
Resolution[a] (Å)	33.70 – 1.60 (1.63 -1.60)	54.35 – 1.96 (2.01 -1.96)
Spacegroup	$P\,4_322$	$P\,4_322$
Cell dimensions	a = 43, b = 43, c = 216.6 Å<br>$\alpha = \beta = \gamma = 90.0°$	a = 42.4, b = 42.4, c = 217.4 Å<br>$\alpha = \beta = \gamma = 90.0°$
No. unique reflections[a]	28203 (1313)	15343 (1046)
Completeness[a] (%)	100.0 (99.9)	98.7 (98.4)
I/σI[a]	24.6 (3.4)	4.6 (0.3)
Rpim[a]	0.016 (0.310)	0.074 (0.973)
CC (1/2)	0.999 (0.977)	0.999 (0.399)
Redundancy[a]	22.4 (22.2)	25.2 (26.8)
Refinement		
No. atoms in refinement	1594	1628
Average B factor (Å^2)	41.0	51.0
R_{fact} (%)	20.8	25.4
R_{free} (%)	25.8	28.9
rms deviation bond[b] (Å)	0.009	0.008
rms deviation angle[b] (°)	1.37	0.99
Molprobity Ramachandran Favored (%)	96.86	96.86
Outlier (%)	0	0.5

[a] Values in brackets show the statistics for the highest resolution shells.

[b] rms indicates root-mean-square.

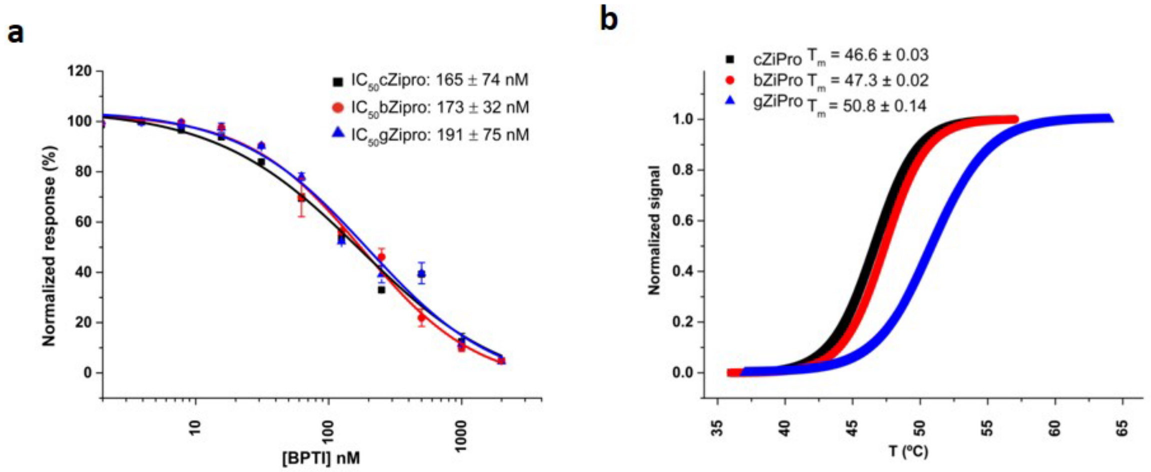

Supplementary Fig. 1 Characterisation of three ZIKV NS2B-NS3 protease constructs cZiPro, bZiPro and gZiPro. (**a**) Inhibition of BPTI against the fluorogenic substrate Bz-nKRR-AMC by cZiPro (black), bZiPro (red), and gZiPro (blue). Protease activity was monitored by fluorescence upon substrate cleavage. Assays were performed in triplicate; error bars represent standard deviation. (**b**) Thermal shift assay of cZiPro (black), bZiPro (red), and gZiPro (blue). cZiPro and bZiPro exhibited comparable thermal stability, with melting temperatures (T_m) of 47.3 °C and 46.6 °C, respectively. In contrast, gZiPro displayed a higher Tm of 50.8 °C, indicating increased thermostability. Data points represent mean values from two independent experiments (n = 2). Error indicate the standard deviation (SD) of the melting temperature (T_m) derived from Boltzmann sigmoidal curve fitting.

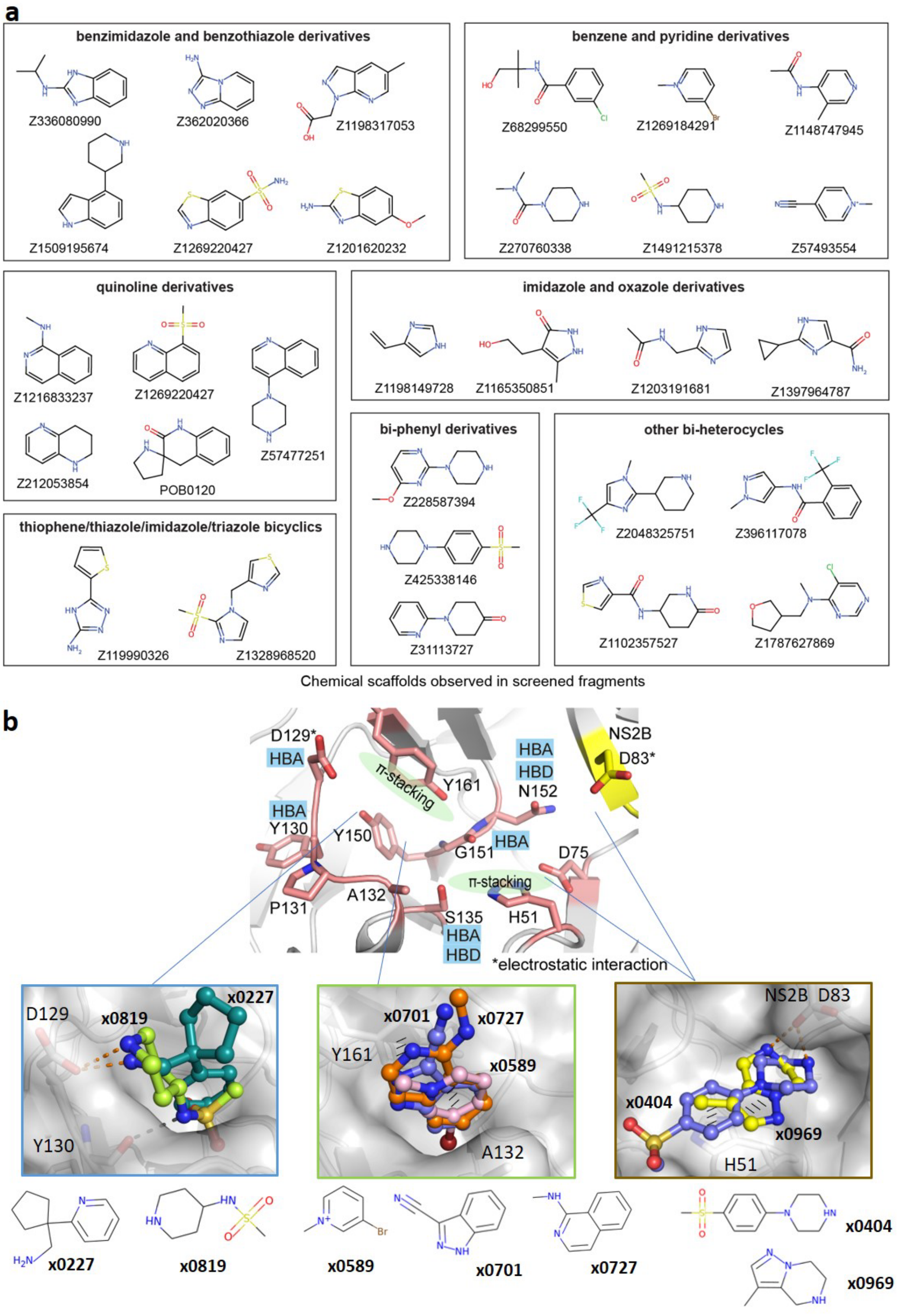

Supplementary Fig. 2 Chemical structures and binding poses of screened fragments. (**a**) Chemical scaffolds observed in bound fragments with fragment examples listed in box. Compound ID is listed at bottom. (**b**) Examples of fragment poses and key interactions observed. Crystal ID is used to represent the pose. Hydrogen bonds are shown as black dashed lines. Electrostatic interaction is shown in orange dashed line.

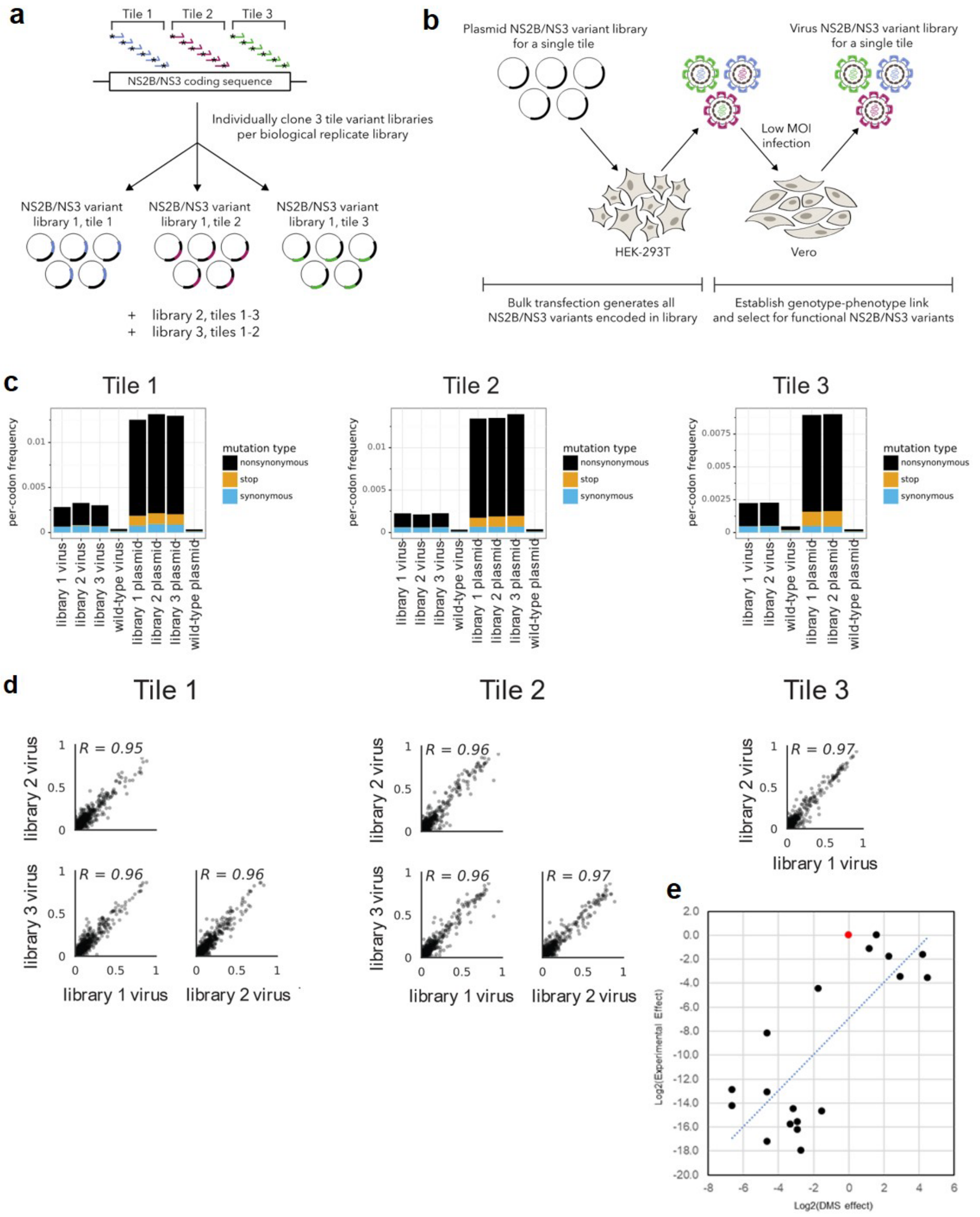

Supplementary Fig. 3 Creation of mutant plasmid libraries and mutant virus of ZIKV NS2B-NS3 protease. (**a**) Using a previously published workflow, we measured the tolerance and preference for all possible amino acid mutations to the entire coding sequence of ZIKV NS2B and NS3 protease over three tiled segments of the genome. Three biological replicates were created for each tile except tile 3 which had only two replicates. (**b**) HEK293T cells were transfected with variants from each tile to create a variant library. The resulting viruses were used to perform a low MOI infection on Vero cells to produce a phenotype-genotype link. (**c**) As expected, stop codons and non-synonymous mutations were purged from the library after passaging. (**d**) There is a high correlation between amino acid preferences measured with replicate deep mutational scanning libraries. (**e**) There is a good correlation between mutation effects measured by DMS and mutation effects measured experimentally (Pearson correlation coefficient of 0.769). Although some mutations were preferred over wildtype (red) in the DMS experiment, no mutations grew better than wildtype (red) at 72 hours post-transfection.

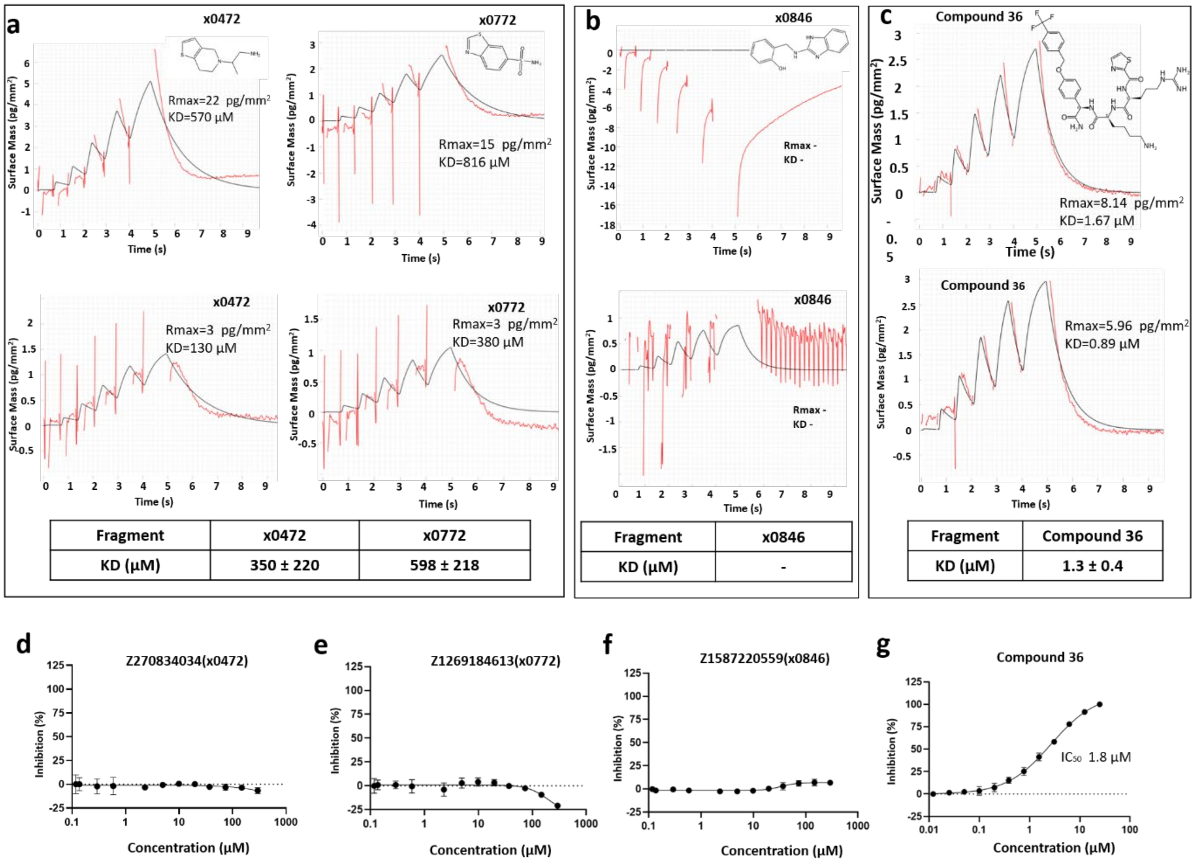

Supplementary Fig. 4 Representative results of screened fragments tested in waveRAPID binding assay and inhibitory activity assay. (**a**) Representative examples of fragments **x0472** and **x0772** showing unreliable binding affinities. (**b**) Representative sensorgrams of fragment **x0846** showing negative and no measurable signal. (**c**) Peptide-hybrid inhibitor compound **36** was used as the positive control, showing a Kd of 1.3 µM. Compound **36** was tested at 1 µM, while fragments were tested at 250 µM. 2D structures of compounds are shown on top. Assays were performed in duplicate; Data are presented as mean values with the standard deviation. (**d**-**f**) Representative examples of screened fragments in fluorescence-dose-response inhibitory activity assay. Fragments **x0472** (**d**), **x0772** (**e**), and **x0846** (**f**) showed no inhibitory activity. (**g**) Positive control compound **36** showing an IC_{50} of 1.8 µM. The IC_{50} plot is shown as representative data from two independent experiments with consistent results. Error bars in IC_{50} plot indicate the standard deviation (SD) from technical duplicates. Source data are provided as a Source Data file.

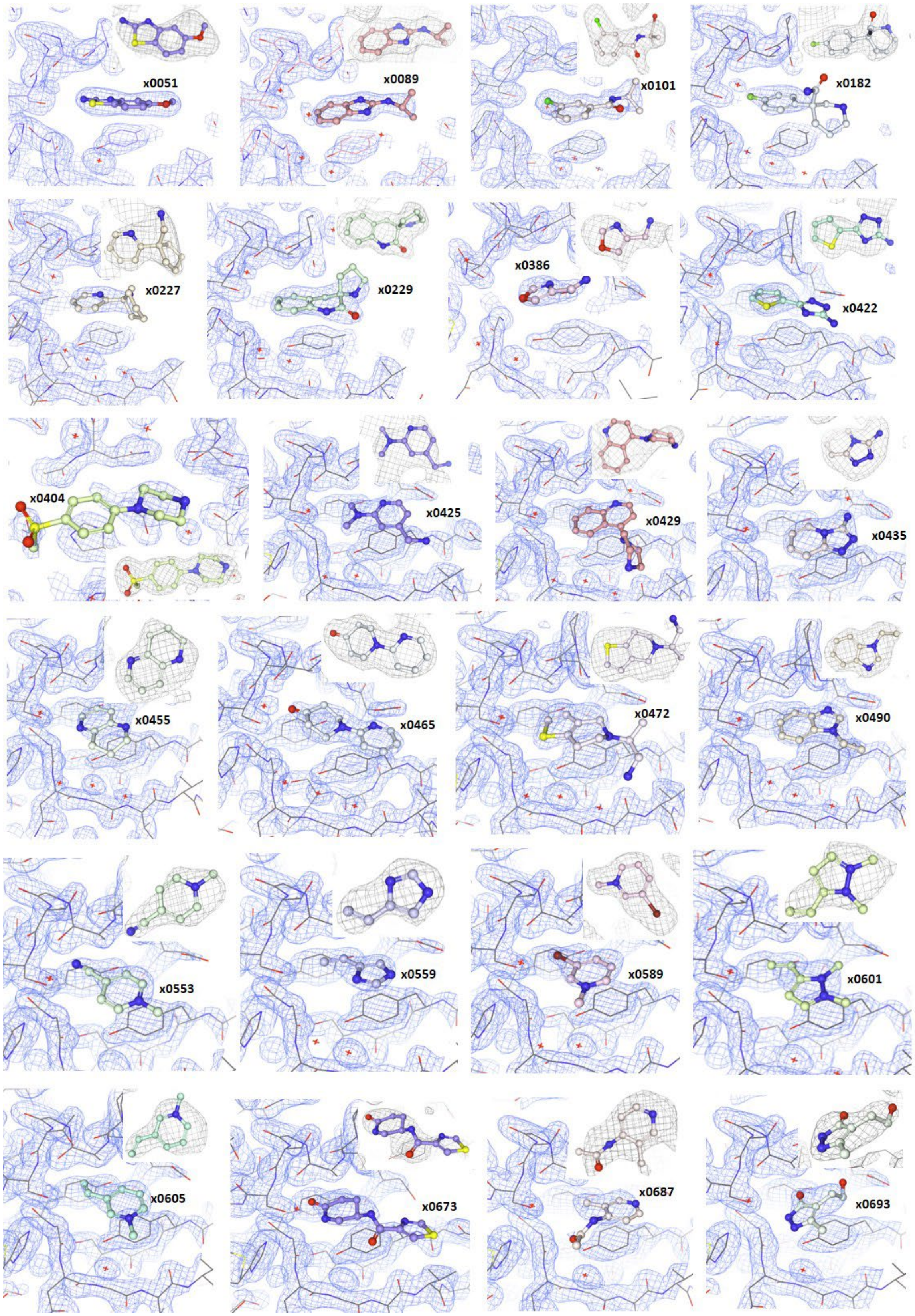

Supplementary Fig.5A 2Fo-Fc map of fragment-bound structures contoured at 1.0 σ. PanDDA event map is shown as grey mesh at 1.0 σ. Crystal ID is used to represent fragment and its binding pose.

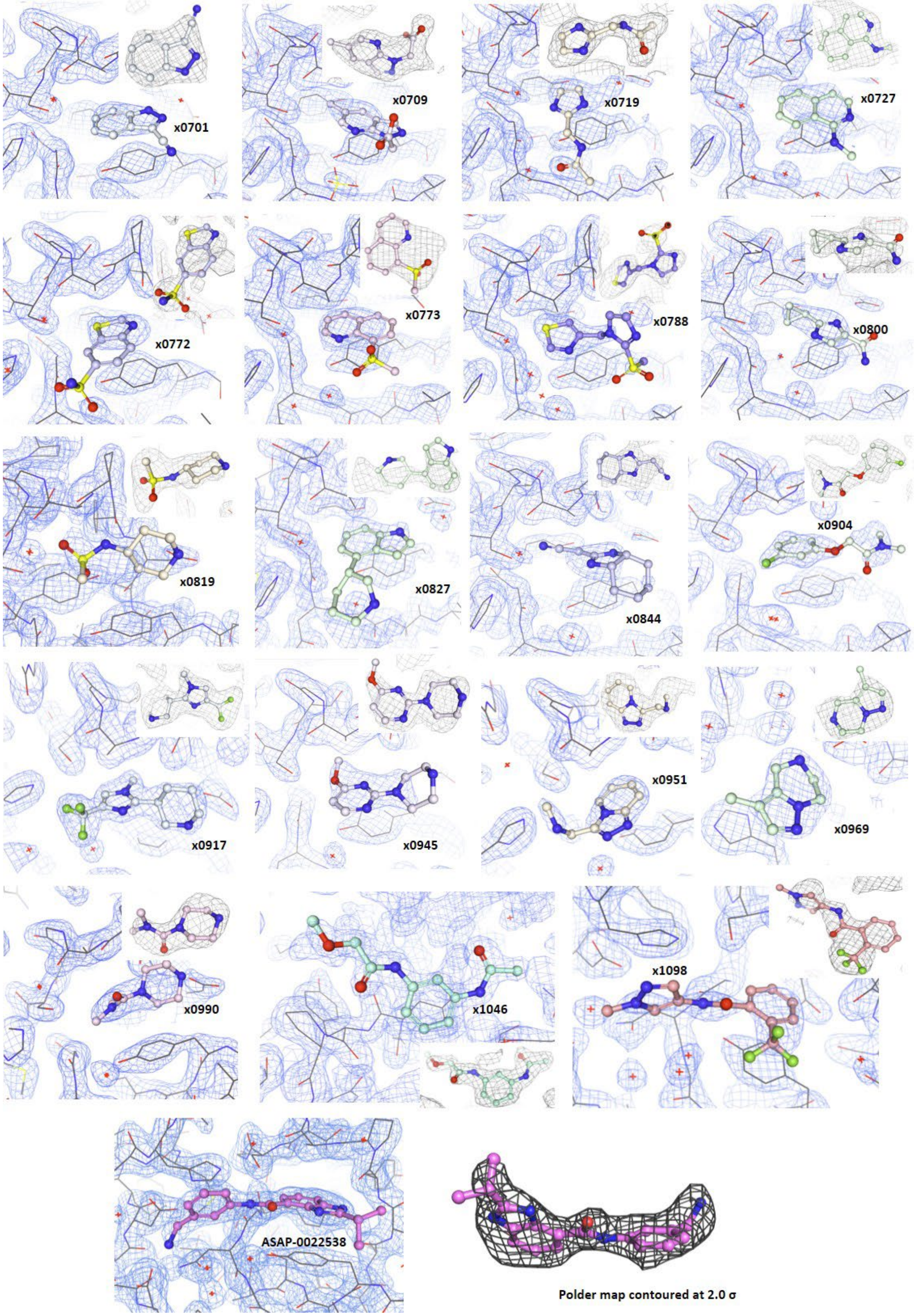

Supplementary Fig.5B 2Fo-Fc map of fragment-bound structures contoured at 1.0 σ. PanDDA event map is shown as grey mesh at 1.0 σ. Crystal ID is used to represent fragment and its binding pose. Polder omit map of ASAP-0022538 is shown as dark grey mesh contoured at 2.0 σ.

Supplementary methods

Supplemental file for DMS experiment

Mutant plasmid libraries were cloned into our previously described single-plasmid reverse genetics system for ZIKV strain MR766[1] (sequence is available at Genbank accession KX830961) using techniques like those described in our previous ZIKV envelope protein DMS paper[2]. In this plasmid, unique XhoI and BstBI restriction sites flank the NS2B-NS3 protease coding sequence. In order to enable efficient cloning into this plasmid backbone, we designed a recipient plasmid in which this 1,119 nucleotide region was replaced by an inert stuffer fragment (sequence available upon request). Any contamination of this vector in our mutant libraries would not result in infectious virus production from the vector.

We created NS2B-NS3 protease codon-mutant libraries using a previously described PCR mutagenesis approach[3]. First, we designed sets of forward and reverse oligos that randomized each codon with an NNK sequence where N is any nucleotide and K is either a G or a T. These primers were designed using a Python script (available at https://github.com/jbloomlab/CodonTilingPrimers) to have an average melting temperature of about ~65 °C[4]. We designed 308 forward and reverse oligo pairs targeting the entire 130 amino acids of NS2B and the 178 amino acids of NS3 comprising its protease domain. To ensure that we could maintain library complexity at all stages of the screen, we subdivided our NS2B-NS3 coding sequencing of interest into three separate tiling DMS library segments, hereinafter referred to simply as "tiles." The first and second tiles each cover 103 mutant codons, and the third tile covers 102 mutant codons. One added benefit to this tile size was that it fit well into a single paired-end Illumina read, which simplified the deep sequencing and analysis. For each tile, we created three individual libraries to be screened as independent biological replicates. Forward and reverse oligos for each tile were synthesized as oPools Oligo Pools (Integrated DNA Technologies). The sequences of these primers are included in the Supplementary Data 2.

We performed the mutagenesis PCR using these primers and the following end primers flanking the NS2B/NS3 protease coding sequence that provide Gibson Assembly overlaps with the XhoI and BstBI restriction sites: 5'-TACCAATCTTGGCTGCTCTAACACCACTAGCTC -3' and 5'-AGTTAGCTGCTTCTTCTTCAGCATCGAGGGTTC -3'. Two rounds of mutagenic PCR were performed in triplicate using a similar approach to previous work[3] with the following adjustments: For the reaction with the forward oPool oligos, 12 ng of WT NS2B/NS3 PCR product was used as template, with 2 µL of the reverse primer at 5 µM, and 2 µL of the forward oPool mixture at 1 µM; For the reaction with the reverse oPool oligos, 12 ng of PCR product was used as template, with 2 µL of 5 µM forward primer, and 1 µL of the "ANN" and "CNN" at 2 µM was used. Due to technical restraints, the reverse NNK pools must be synthesized as two separate libraries, one representing all "ANN" mutants and one representing all "CNN" mutants. These were used in a standard PCR reaction using Q5 High-Fidelity DNA Polymerase (New England Biolabs, Ipswich, MA) in a final volume of 30 µL. The PCR template was amplified using the program of (1) 98 °C for 2:00; (2) 98

°C for 0:10; (3) 70 °C for 0:01; (4) cool at 0.5 °C/sec to 50 °C and hold for 0:30; (5) 70 °C for 1:30; 6) Go to (2) (3 total cycles); (7) 70 °C 2:00; (8) 4 °C hold. Then, the forward and reverse PCR reactions were diluted 1:4, and 4 µL of these dilutions were used in a second round of PCR, with 2 µL of the forward and reverse primers above at 5 µM in a 30 µL PCR reaction. The PCR program was identical to that above except it was used for 20 total cycles. The resulting PCR products were gel purified using an E.Z.N.A. Gel Extraction Kit (Omega Biotek, Norcross, GA). After XhoI and BstBI digestion and purification of the recipient vector plasmid, mutagenized NS2B-NS3 protease amplicons were cloned into the recipient MR766 genome using Gibson assembly and transformed into 10-beta electrocompetent E. coli (NEB). Transformants were plated on LB supplemented with ampicillin for counting colony numbers and in bottle culture of LB supplemented with carbenicillin for full library growth[5, 6]. To help ensure higher plasmid stability, the bacterial growth steps were performed at 30°C. Each codon mutant library was prepared by HiSpeed Maxiprep (Qiagen) from transformants harvested from each bottle culture.

Rescue and screening of ZIKV NS2B-NS3 protease mutant libraries.

ZIKV NS2B/NS3 protease DMS libraries were produced by transfecting plasmids into HEK 293T cells using a protocol to maintain library complexity as previously reported[2]. Wild type virus was rescued in parallel as a control. Briefly, prior to transfection, poly-lysine coated 6-well plates were seeded overnight with 4 × 10^5 293T cells. For each library replicate, all wells of a 6-well plate were transfected with 2 µg of plasmid DNA per well and the TransIT-LT1 transfection reagent (Mirus Bio, Madison WI) following the manufacturer's recommendations. The viral supernatants were collected at day 2 and 3 post transfection, pooled, centrifuged to remove cellular debris, and stored at −80 °C.

Titration of infectious viruses and ZIKV NS2B-NS3 libraries.

The infectious titers of rescued wild type virus and mutant virus libraries were determined by immunostaining with the pan-flavivirus E protein reactive 4G2 antibody and flow cytometry, as previously described[7]. Briefly, one day prior to infection, 24-well plates were seeded at a density of 1 x 10^5 cells/well. The next day, 250 uL of serially diluted transfection supernatants in DMEM with 3% FBS was added to each well (in duplicate). At 24 hours post infection, at which time initial infection events would be detectable but secondary infections would not be, cells were fixed in 4% paraformaldehyde and stained with 4G2 antibodies and goat-anti-human secondary antibody conjugated to Alexa Fluor 647 (Thermo Fisher Scientific, Waltham, MA). Data were acquired on an Attune flow cytometer (Thermo Fisher Scientific, Waltham, MA) and analyzed using FlowJo (Tree Star, USA). Only the viral dilutions leading to less than 20% of infected cells were used to calculate the infectious titers. Infectious units per mL were calculated using the following formula: "percentage of infectious events x number of cells / volume (mL) of viral inoculum".

Deep sequencing using barcoded-subamplicon sequencing

Total RNA for each tile within the NS2B/NS3 protease coding sequence was reverse transcribed using AccuScript High Fidelity Reverse Transcriptase (Agilent Technologies, La Jolla, CA) with

primers for tile 1 (5’ - TTACTCACAAGGAGTGGGAAG - 3’ and 5’ - CATGCCACAGATGGCCATCAG - 3’), tile 2 (5’ – GAGATCATACTCAAGGTGGTC - 3’ and 5’ - AGGCCCACAGTATGACACCAA - 3’), and tile 3 (5’ – TGGGGGGATGTCAAGCAGGAC - 3’ and 5’ - CAGCATCGAGGGTTCGAAACA - 3’). Following the manufacturer’s instructions, we used 1 ug of RNA for each sample. To obtain high sequencing accuracy, we followed the barcoded-subamplicon sequencing approach described[8] (see also https://jbloomlab.github.io/dms_tools2/bcsubamp.html). We generated amplicons for each of the NS2B/NS3 protease tiles using the same RT primers listed above, followed by two barcoding PCRs. For the amplicon PCR, we used 2 µL of the RT reaction or 10ng for amplification of the plasmid libraries. The cycling program used for the amplicon PCR was: (1) 98 °C for 2:00; (2) 98°C for 0:10; (3) 70 °C for 0:01; (4) cool at 0.5°C/sec to 50°C and hold for 0:30; (5) 70 °C for 0:30; (6) Go to (2) (20 total cycles); (7) 70 °C 2:00; (8) 4 °C hold. The first primer used for amplification adds an N8 randomized barcode and the second primer adds Illumina adapter sequences. Round 1 PCR reactions were set up using Q5 Polymerase, 4 ng of amplicon PCR product, 2 µL of forward primer diluted to 5 µM, and 2 µL of reverse primer diluted to 5 µM. The primers used in Round 1 PCR for tile 1 (5’ - CTTTCCCTACACGACGCTCTTCCGATCTNNNNNNNNTTACTCACAAGGAGTGGGAAG - 3’ and 5’ - GGAGTTCAGACGTGTGCTCTTCCGATCTNNNNNNNNCATGCCACAGATGGCCATCAG - 3’), tile 2 (5’ - CTTTCCCTACACGACGCTCTTCCGATCTNNNNNNNNGAGATCATACTCAAGGTGGTC -3’ and 5’ - GGAGTTCAGACGTGTGCTCTTCCGATCTNNNNNNNNAGGCCCACAGTATGACACCAA - 3’), and for tile 3 (5’ - CTTTCCCTACACGACGCTCTTCCGATCTNNNNNNNNTGGGGGGATGTCAAGCAGGAC - 3’ and 5’ - GGAGTTCAGACGTGTGCTCTTCCGATCTNNNNNNNNCAGCATCGAGGGTTCGAAACA - 3’) are listed. The cycling program used for the Round 1 PCR was: (1) 98 °C for 2:00; (2) 98 °C for 0:10; (3) 70 °C for 0:01; (4) cool at 0.5 °C/sec to 50 °C and hold for 0:30; (5) 70 °C for 0:30; (6) Go to (2) (9 total cycles); (7) 70 °C 2:00; (8) 95 °C for 1:00; (9) 4 °C hold. The final denaturation step ensures that double-stranded DNA molecules entering Round 2 PCR will contain two uniquely barcoded variants.

Amplicons were diluted and used as template for a second round of PCR using ~2× 10^5 single-stranded template molecules per sample. The primers used for Round 2 PCR add sample-specific indices and the Illumina cluster-generating sequences. The PCRs used a universal forward primer (5’ - AATGATACGGCGACCACCGAGATCTACACTCTTTCCCTACACGACGCTCTTCCGATCT - 3’) and a sample-specific reverse primer that allows for indexing of the reads during sequencing with general format of (5’ - CAAGCAGAAGACGGCATACGAGATxxxxxxxxGTGACTGGAGTTCAGACGTGTGCTCTTCCGATCT -3’) where x represents an index-specific nucleotide. The Round 2 PCR reactions contained Q5 Polymerase, Round 1 DNA diluted to 2× 10^5 ssDNA molecules per subamplicon, 4 µL of forward primer diluted to 5 µM, and 4 µL of reverse primer diluted to 5 µM. The cycling program used for Round 2 PCR was: (1) 98 °C for 2:00; (2) 98 °C for 0:10; (3) 70 °C for 0:01; (4) cool at 0.5 °C/sec to 50 °C and hold for 0:30; (5) 70 °C for 0:30; (6) Go to (2) (24 total cycles); (7) 70 °C 2min; (8) 4 °C Hold. After each PCR step (amplicon, Round 1 and Round 2), amplicons were purified using AMPure XP beads (Beckman Coulter, Brea, CA) (1x bead-to-sample volume for amplicon, and 1.6x bead-to-sample volume for Round 1 and Round 2). DNA concentration was quantified using

Quant-iT PicoGreen dsDNA Assay Kit (Thermo Fisher Scientific, Waltham, MA). After barcoding, the Round 2 subamplicons were pooled, size selected by gel extraction, purified with an E.Z.N.A. Gel Extraction Kit (Omega Biotek, Norcross, GA), and purified with AMPure XP beads (1.6x bead-to-sample volume). Subamplicons were sequenced with an Illumina HiSeq2500 using 2 × 250 bp paired-end reads in Rapid Run mode. The raw deep sequencing data have been deposited in the Sequence Read Archive as BioProject PRJNA1125458.

ZIKV Gluc validation of DMS results

To validate the predicted fitness of mutants from the DMS screen, mutants were cloned into a pACYC177 plasmid. The expression plasmid pACYC177 MR766 contains a ZIKV MR766 genome with a Gaussia luciferase inserted between NS1 and NS2A under the control of a T7 promoter. These plasmids were linearized with ScaI and purified with a Zymo DCC-5 column. The linearized DNA was used as a template in a Promega T7 Express In vitro transcription reaction, with the addition of m7g cap. The reaction was purified with the EZNA RNA Kit and the products were aliquoted to 5µg per tube and stored in the –80 °C.

For each electroporation, Huh7.5 cells that were washed twice in ice cold PBS were resuspended at 1.5x10^7 cells/mL. Approximately 400µL (6x10^6 cells) were added to Eppendorf tubes and mixed with 5µg of RNA. The mixture was transferred to a 1mm gap cuvette and electroporated with the following conditions. Cells were rested for 10 minutes in the cuvettes, then transferred to 10 mL of pre-warmed DMEM with 3% FBS.

Electroporated cells were seeded at a density of 80,000-100,000 cells per well in a 24-well plate, along with 100,000 non-electroporated cells to enhance spread of virus in the well. Cells were harvested 3 days post electroporation and luciferase activity was measured using a Renilla luciferase assay.

Production of recombinant bZipro ZIKV NS2B-NS3

The bZiPro plasmid encoding the recombinant ZIKV protease was commercially obtained from Addgene (plasmid #86846). This construct encodes residues 45–96 of the NS2B cofactor and residues 1–177 of the NS3 protease domain, expressed independently and non-covalently assembled in the cytoplasm to form the active NS2B-NS3pro complex, as originally described by Phoo et al[9].

Escherichia coli BL21 (DE3) cells harboring the bZiPro plasmid were cultured in LB medium supplemented with 50 mM potassium phosphate buffer (pH 7.4) and 2.5% glycerol. Upon reaching an OD_{600} of 0.8, protein expression was induced with 1 mM IPTG for 16 hours at 18 °C. Cells were harvested by centrifugation at 4,000 × g for 40 min at 4 °C and resuspended in lysis buffer (buffer A: 500 mM NaCl, 20 mM PBS pH 8.0, 10 mM imidazole, 2 mM β-mercaptoethanol, 5% glycerol) supplemented with 0.5 mg/mL lysozyme and 0.1 mg/mL Benzonase. Lysis was performed by sonication, and the lysate was clarified by centrifugation at 13,000 × g for 1 hour at 4 °C.

The supernatant was loaded onto a 5 mL HisTrap HP column (GE Healthcare) for immobilized metal affinity chromatography (IMAC). After washing with lysis buffer (buffer A), the protein was eluted using a 10–30% gradient of elution buffer (buffer B: 500 mM NaCl, 20 mM PBS pH 8.0, 300 mM imidazole, 2 mM β-mercaptoethanol, 5% glycerol). The 6×His-GST tag was removed by thrombin digestion during overnight dialysis (16 h at 4 °C) against 2 L of buffer A. The digested sample was subjected to reverse IMAC to remove uncleaved protein and free tag and subsequently passed through a Benzamidine column to remove residual thrombin. Final purification was performed by size-exclusion chromatography using a HiLoad 16/60 Superdex 75 column (GE Healthcare) equilibrated with gel-filtration buffer (20 mM HEPES pH 7.5, 150 mM NaCl, 2 mM DTT, 5% glycerol).

Production of recombinant gZipro ZIKV NS2B-NS3

The plasmid containing the gZipro sequence of the recombinant ZIKV protease, corresponding to residues 45-96 of the NS2B cofactor covalently linked to residues 1-177 of the NS3 protease domain by a glycine-rich linker [G4SG4] using Gibson assembly, to form the active NS2B-NS3^{pro} complex as described by Phoo et al[9].

Escherichia coli Rosetta 2 (DE3) cells harboring the gZiPro plasmid encoding the recombinant ZIKV protease were grown in Terrific Broth (TB) medium at 37°C until reaching an optical density at 600 nm (OD_{600}) of 1.0. Protein expression was induced with 0.5 mM IPTG (β-D-1-thiogalactopyranoside) for 16 hours at 18°C. Cells were harvested by centrifugation at 4,000 × g for 40 minutes at 4°C and resuspended in lysis buffer (300 mM NaCl, 50 mM Tris-HCl pH 8.0, 30 mM imidazole, 10% glycerol) supplemented with 0.5 mg/mL lysozyme and 0.1 mg/mL Benzonase. Lysis was performed by sonication, and the clarified lysate was obtained by centrifugation at 13,000 × g for 1 hour at 4 °C.

The supernatant was subjected to immobilized metal affinity chromatography (IMAC) using a 5 mL HisTrap HP column (GE Healthcare). After washing with lysis buffer, the protein was eluted with a gradient of elution buffer containing 300 mM NaCl, 50 mM Tris-HCl pH 8.0, 300 mM imidazole, 10% glycerol. To remove the N-terminal 6×His-GST tag, TEV protease was added, and the mixture was dialyzed overnight (16 h at 4°C) against 2 L of lysis buffer. Following dialysis, reverse IMAC was performed to remove uncleaved protein and free tag. The cleaved protein was further purified by size-exclusion chromatography on a HiLoad 16/60 Superdex 75 column (GE Healthcare) in buffer of 20 mM HEPES pH 7.5, 500 mM NaCl, 5% glycerol. Final aliquots were flash-frozen in liquid nitrogen and stored at –80 °C.

Enzyme activity and characterization

The proteolytic activity of NS2B-NS3pro was assessed by monitoring the fluorescence generated upon cleavage of the fluorogenic substrate BZ-nKRR-AMC (International Peptides), which releases 7-amino-4-methylcoumarin (AMC) upon hydrolysis[10]. Reactions were performed in black

384-well Corning® microplates using a reaction buffer composed of 20 mM Tris-HCl pH 8.5, 10% glycerol, and 0.01% Triton X-100. The protease was added at a final concentration of 5 nM to wells prefilled with reaction buffer, followed by the addition of test compounds at 10 µM. After a 15-minute pre-incubation at 37 °C, the substrate was added to a final concentration of 30 µM. The reaction was carried out at 37 °C, and fluorescence was recorded every minute for 25 minutes using excitation and emission wavelengths of 380 nm and 460 nm, respectively.

Differential Scanning Fluorimetry (DSF)

The thermal stability of the recombinant NS2B-NS3 protease constructs was assessed by determining melting temperatures (Tm) using Differential Scanning Fluorimetry (DSF). Reactions were performed with 5 µM protein in buffer containing 25 mM HEPEs pH 7.5, 150 mM NaCl, 0.5 mM TCEP, and 5% glycerol, in the presence of 5× SYPRO Orange dye (Sigma-Aldrich). DSF measurements were carried out on a Stratagene Mx3005P real-time PCR system (Agilent Technologies), with a temperature gradient from 25 °C to 75 °C at a ramp rate of 1 °C per cycle. Fluorescence was monitored using excitation and emission wavelengths of 490 nm and 530 nm, respectively. Fluorescence curves were analyzed in Origin 9.0 software (OriginLab), and Tm values were obtained by fitting the data to a Boltzmann sigmoidal model.

Reference

(1) Schwarz, M. C.; Sourisseau, M.; Espino, M. M.; Gray, E. S.; Chambers, M. T.; Tortorella, D.; Evans, M. J. Rescue of the 1947 Zika Virus Prototype Strain with a Cytomegalovirus Promoter-Driven cDNA Clone. *mSphere* **2016**, *1* (5). DOI: 10.1128/mSphere.00246-16 From NLM PubMed-not-MEDLINE.
(2) Sourisseau, M.; Lawrence, D. J. P.; Schwarz, M. C.; Storrs, C. H.; Veit, E. C.; Bloom, J. D.; Evans, M. J. Deep Mutational Scanning Comprehensively Maps How Zika Envelope Protein Mutations Affect Viral Growth and Antibody Escape. *J Virol* **2019**, *93* (23). DOI: 10.1128/JVI.01291-19 From NLM Medline.
(3) Bloom, J. D. An experimentally determined evolutionary model dramatically improves phylogenetic fit. *Mol Biol Evol* **2014**, *31* (8), 1956-1978. DOI: 10.1093/molbev/msu173 From NLM Medline.
(4) Dingens, A. S.; Haddox, H. K.; Overbaugh, J.; Bloom, J. D. Comprehensive Mapping of HIV-1 Escape from a Broadly Neutralizing Antibody. *Cell Host Microbe* **2017**, *21* (6), 777-787 e774. DOI: 10.1016/j.chom.2017.05.003 From NLM Medline.
(5) Kriegler, M. *Gene Transfer and Expression: A Laboratory Manual.*; 1990.
(6) Hanahan, D.; Jessee, J.; Bloom, F. R. Plasmid transformation of Escherichia coli and other bacteria. *Methods Enzymol* **1991**, *204*, 63-113. DOI: 10.1016/0076-6879(91)04006-a From NLM Medline.
(7) Crill, W. D.; Chang, G. J. Localization and characterization of flavivirus envelope glycoprotein cross-reactive epitopes. *J Virol* **2004**, *78* (24), 13975-13986. DOI: 10.1128/JVI.78.24.13975-13986.2004 From NLM Medline.
(8) Doud, M. B.; Bloom, J. D. Accurate Measurement of the Effects of All Amino-Acid Mutations on Influenza Hemagglutinin. *Viruses* **2016**, *8* (6). DOI: 10.3390/v8060155 From NLM Medline.
(9) Phoo, W. W.; Li, Y.; Zhang, Z. Z.; Lee, M. Y. Q.; Loh, Y. R.; Tan, Y. B.; Ng, E. Y.; Lescar, J.; Kang, C. B.; Luo, D. H. Structure of the NS2B-NS3 protease from Zika virus after self-cleavage. *Nature Communications* **2016**, *7*. DOI: ARTN 1341010.1038/ncomms13410.

(10) Fernandes, R. S.; Noske, G. D.; Gawriljuk, V. O.; de Oliveira, K. I. Z.; Godoy, A. S.; Mesquita, N.; Oliva, G. High-throughput Antiviral Assays to Screen for Inhibitors of Zika Virus Replication. *J Vis Exp* **2021**, (176). DOI: 10.3791/62422 From NLM Medline.
