# Supplementary material for: Combined crystallographic fragment screening and deep mutational scanning enable discovery of Zika virus NS2B-NS3 protease inhibitors
Source: Nat Commun. 2025 Oct 8;16:8930. doi: 10.1038/s41467-025-63602-z (PMC12508225; doi:10.1038/s41467-025-63602-z)
Supplement: Supplementary file 3 — Description of Additional Supplementary Files [file 41467_2025_63602_MOESM3_ESM.pdf]

File Name: Supplementary Data 1

Description: This file contains the SMILES of screened fragments and tables of structural and refinement statistics for fragment-bound structures in this study.

File Name: Supplementary Data 2

Description: This file contains all primers used for DMS experiment in this study.
