# Supplementary material for: Combined crystallographic fragment screening and deep mutational scanning enable discovery of Zika virus NS2B-NS3 protease inhibitors
Source: Nat Commun. 2025 Oct 8;16:8930. doi: 10.1038/s41467-025-63602-z (PMC12508225; doi:10.1038/s41467-025-63602-z)
Supplement: Supplementary file 6 — Reporting Summary [file 41467_2025_63602_MOESM6_ESM.pdf]

nature portfolio

Corresponding author(s): Xiaomin Ni, Matthew J. Evans, Frank von Delft

Last updated by author(s): Aug 6, 2025

Reporting Summary

Nature Portfolio wishes to improve the reproducibility of the work that we publish. This form provides structure for consistency and transparency in reporting. For further information on Nature Portfolio policies, see our Editorial Policies and the Editorial Policy Checklist.

Statistics

For all statistical analyses, confirm that the following items are present in the figure legend, table legend, main text, or Methods section.

n/a	Confirmed	
☐	☒	The exact sample size (*n*) for each experimental group/condition, given as a discrete number and unit of measurement
☐	☒	A statement on whether measurements were taken from distinct samples or whether the same sample was measured repeatedly
☒	☐	The statistical test(s) used AND whether they are one- or two-sided<br>*Only common tests should be described solely by name; describe more complex techniques in the Methods section.*
☒	☐	A description of all covariates tested
☒	☐	A description of any assumptions or corrections, such as tests of normality and adjustment for multiple comparisons
☒	☐	A full description of the statistical parameters including central tendency (e.g. means) or other basic estimates (e.g. regression coefficient) AND variation (e.g. standard deviation) or associated estimates of uncertainty (e.g. confidence intervals)
☒	☐	For null hypothesis testing, the test statistic (e.g. *F*, *t*, *r*) with confidence intervals, effect sizes, degrees of freedom and *P* value noted<br>*Give P values as exact values whenever suitable.*
☒	☐	For Bayesian analysis, information on the choice of priors and Markov chain Monte Carlo settings
☒	☐	For hierarchical and complex designs, identification of the appropriate level for tests and full reporting of outcomes
☐	☒	Estimates of effect sizes (e.g. Cohen's *d*, Pearson's *r*), indicating how they were calculated

Our web collection on statistics for biologists contains articles on many of the points above.

Software and code

Policy information about availability of computer code

Data collection	The software used is fully described in the Methods.
Data analysis	All used softwares have been described in Method or Maintext. Notebooks are made available on github.https://github.com/jbloomlab/ZIKV_DMS_NS3_EvansLab.

For manuscripts utilizing custom algorithms or software that are central to the research but not yet described in published literature, software must be made available to editors and reviewers. We strongly encourage code deposition in a community repository (e.g. GitHub). See the Nature Portfolio guidelines for submitting code & software for further information.

Data

Policy information about availability of data

All manuscripts must include a data availability statement. This statement should provide the following information, where applicable:

- Accession codes, unique identifiers, or web links for publicly available datasets
- A description of any restrictions on data availability
- For clinical datasets or third party data, please ensure that the statement adheres to our policy

Construct cZiPro has been deposited to Addgene https://www.addgene.org/228663/. cZiPro is designed based on PDB structure 5GPI [https://www.rcsb.org/structure/5GPI]. Crystallographic coordinates and structure factors for cZiPro crystal structure and ASAP-0022538-bound structure have been deposited in the PDB with the accession codes 8PN6 [https://www.rcsb.org/structure/8PN6] and 7I9O [https://www.rcsb.org/structure/7I9O], respectively. PDB codes of crystallographic

fragment binders are listed in Crystallographic Supplementary Table. Source data are provided with this paper. The raw deep sequencing data have been deposited in the Sequence Read Archive as BioProject PRJNA1125458. Results of deep sequencing data are available on GitHub at https://github.com/jbloomlab/ZIKV_DMS_NS3_EvansLab. Source data are provided with this paper.

Research involving human participants, their data, or biological material

Policy information about studies with human participants or human data. See also policy information about sex, gender (identity/presentation), and sexual orientation and race, ethnicity and racism.

Reporting on sex and gender	N/A
Reporting on race, ethnicity, or other socially relevant groupings	N/A
Population characteristics	N/A
Recruitment	N/A
Ethics oversight	N/A

Note that full information on the approval of the study protocol must also be provided in the manuscript.

Field-specific reporting

Please select the one below that is the best fit for your research. If you are not sure, read the appropriate sections before making your selection.

☒ Life sciences ☐ Behavioural & social sciences ☐ Ecological, evolutionary & environmental sciences

For a reference copy of the document with all sections, see nature.com/documents/nr-reporting-summary-flat.pdf

Life sciences study design

All studies must disclose on these points even when the disclosure is negative.

Sample size	The sample size of fragment screen is a typical size used in crystallographic fragment screen.<br>Sample size for DMS was based on prior experiments with similar assays. Each was sufficient to provide statistically significant results.
Data exclusions	Crystallographic Data exclusion was done algorithmically- data with poor quality (i.e. low resolution >2.8A) was excluded. No data exclusions for other experiments
Replication	Replications for each relevant experiment were mentioned in legends and Method. Typically all the experiments were conducted in duplicates-triplicates (stated in legends). Replication for fragment related assay was not attempted, as these were screening experiments.
Randomization	No randomisation was conducted
Blinding	No blinding was conducted

Reporting for specific materials, systems and methods

We require information from authors about some types of materials, experimental systems and methods used in many studies. Here, indicate whether each material, system or method listed is relevant to your study. If you are not sure if a list item applies to your research, read the appropriate section before selecting a response.

Materials & experimental systems

n/a	Involved in the study	
☐	☒	Antibodies
☐	☒	Eukaryotic cell lines
☒	☐	Palaeontology and archaeology
☒	☐	Animals and other organisms
☒	☐	Clinical data
☒	☐	Dual use research of concern
☒	☐	Plants

Methods

n/a	Involved in the study	
☒	☐	ChIP-seq
☒	☐	Flow cytometry
☒	☐	MRI-based neuroimaging

Antibodies

Antibodies used	humanized monoclonal antibody D1-4G2-4-15 (4G2) (Absolute Antibody, Oxford, UK)
Validation	The specificity of 4G2 for the envelope (E) protein of ZIKV has been validated in multiple published studies and by the supplier. We included internal controls to verify antibody specificity in our experiment. These controls included naïve (uninfected) cells, which consistently showed no staining, confirming that signal was specific to cells expressing the ZIKV E protein.

Eukaryotic cell lines

Policy information about cell lines and Sex and Gender in Research

Cell line source(s)	Human embryonic kidney derived 293T cells, Human liver derived Huh-7.5 cells
Authentication	None of the cell lines are authenticated
Mycoplasma contamination	Clean of mycoplasma
Commonly misidentified lines (See ICLAC register)	No known commonly misidentified cell lines. 293T cells are used for the production of recombinant ZIKV stocks. Huh-7.5 cells are used for ZIKV infection assays.

Plants

Seed stocks	n/a
Novel plant genotypes	n/a
Authentication	n/a
